# Bird Flu: A Virus of Our Own Hatching

**DOI:** 10.3201/eid1311.070787

**Published:** 2007-11

**Authors:** David J. Sencer

**Affiliations:** * Atlanta, Georgia, USA

**Keywords:** avian flu, H5N1, H6N1, book review

Bird Flu: A Virus of Our Own Hatching, by Michael Gerber, MD, is written for a nonprofessional audience. A professional audience would quickly put it aside for more factually correct sources of information.

Dr. Gerber is the director of Public Health and Animal Agriculture at the Humane Society of the United States. Much of the book is devoted to criticism of the commercial farming of birds and other animals; agricultural practices are blamed for the threat of pandemic influenza. He neglects the fact that most of the cases of human infection with influenza A (H5N1) have come from family farms in Asia, rather than the large commercial ventures.

The science relating to the current subtype H6N1 is changing so rapidly that any book is out-of-date by the time it is published. The book contains 90 pages of references, mostly from the popular press. Few current peer-reviewed sources are cited.

The need for authoritative information on avian influenza (H5N1) for the lay public is great, but unfortunately, this book does not meet that need. It focuses heavily on doomsday scenarios and offers little in terms of practical advice to the public. For those interested in the book, it can be found online at www.birdflubook.com.

